# Blood Mercury Concentrations in CHARGE Study Children with and without Autism

**DOI:** 10.1289/ehp.0900736

**Published:** 2009-10-19

**Authors:** Irva Hertz-Picciotto, Peter G. Green, Lora Delwiche, Robin Hansen, Cheryl Walker, Isaac N. Pessah

**Affiliations:** 1 Department of Public Health Sciences; 2 Children’s Center for Environmental Health and Disease Prevention; 3 MIND (Medical Investigations of Neurodevelopmental Disorders) Institute; 4 Department of Civil and Environmental Engineering; 5 Department of Pediatrics; 6 Department of Obstetrics and Gynecology and; 7 Department of Molecular Biosciences, University of California–Davis, Davis, California, USA

**Keywords:** autism, autism spectrum disorders, child development, dental amalgams, developmental delay, fish, mercury, metabolism, metals

## Abstract

**Background:**

Some authors have reported higher blood mercury (Hg) levels in persons with autism, relative to unaffected controls.

**Objectives:**

We compared blood total Hg concentrations in children with autism or autism spectrum disorder (AU/ASD) and typically developing (TD) controls in population-based samples, and determined the role of fish consumption in differences observed.

**Methods:**

The Childhood Autism Risk from Genetics and the Environment (CHARGE) Study enrolled children 2–5 years of age. After diagnostic evaluation, we analyzed three groups: AU/ASD, non-AU/ASD with developmental delay (DD), and population-based TD controls. Mothers were interviewed about household, medical, and dietary exposures. Blood Hg was measured by inductively coupled plasma mass spectrometry. Multiple linear regression analysis was conducted (*n* = 452) to predict blood Hg from diagnostic status controlling for Hg sources.

**Results:**

Fish consumption strongly predicted total Hg concentration. AU/ASD children ate less fish. After adjustment for fish and other Hg sources, blood Hg levels in AU/ASD children were similar to those of TD children (*p* = 0.75); this was also true among non-fish eaters (*p* = 0.73). The direct effect of AU/ASD diagnosis on blood Hg not through the indirect pathway of altered fish consumption was a 12% reduction. DD children had lower blood Hg concentrations in all analyses. Dental amalgams in children with gum-chewing or teeth-grinding habits predicted higher levels.

**Conclusions:**

After accounting for dietary and other differences in Hg exposures, total Hg in blood was neither elevated nor reduced in CHARGE Study preschoolers with AU/ASD compared with unaffected controls, and resembled those of nationally representative samples.

Autism is a pervasive developmental disorder characterized by deficits in reciprocal social interactions and communication and by stereotyped, repetitive behaviors or a restricted range of interests. Autism spectrum disorders (ASDs) also include Asperger’s syndrome, a milder form of autism, and “pervasive developmental delay–not otherwise specified” [*Diagnostic and Statistical Manual of Mental Disorders*, 4th ed. (American Psychiatric Association 2000)]. Established risk factors include male sex (Fombonne 2003), higher maternal and/or paternal age (Durkin et al. 2008), and family history of ASD (Piven et al. 1991). Heritability is high (Ritvo et al. 1985), and genomewide association studies indicate linkage with regions on every chromosome, suggesting a large number of genes may confer heightened autism susceptibility (Spence et al. 1985; Ylisaukko-oja et al. 2006). Two decades ago, Folstein and Rutter (1988) concluded: “Quite often it is not autism itself that is inherited but rather some genetic abnormality of language or sociability that interacts with other factors to produce autism.” With discordance in up to 40% of monozygotic twin pairs, differential gene expression in autism cases compared with unaffected individuals (Gregg et al. 2008; Kelleher and Bear 2008), and epigenetic variation in DNA methylation (LaSalle 2007), environmental influences plausibly act both in concert with and independently of heritable factors.

Because of its known neurotoxicity, mercury has drawn particular attention in relation to autism. Investigations have compared measurements of Hg in blood, hair, or urine in children with versus without autism. Measurements on specimens collected before a diagnosis could, at least theoretically, shed light on the causal hypothesis. One group reported lower concentrations in the first haircut from children with autism (Holmes et al. 2003). However, the mean hair concentration in their control series was unusually high: 16-fold greater than the mean from the nationally representative National Health and Nutrition Examination Survey (NHANES) sample of children 1–5 years of age. In fact, the vast majority of measurements on the controls were above the 95th percentile in the NHANES sample, whereas the concentrations in children with the greatest severity of autism were very close to the national average (McDowell et al. 2004). These issues, combined with absence of information regarding study design and laboratory quality control and assurance, raise concerns about the validity of the findings.

Studies conducting measurements in specimens collected postdiagnosis do not directly address the role of Hg as a causal factor in autism but may provide evidence about recent exposure or about toxicokinetics, including absorption, metabolism, distribution, and excretion. Higher hair concentrations of Hg, lead, and uranium were reported for 40 boys with autism compared with 40 unaffected boys (mean age, 4.2–4.3 years) (Fido and Al-Saad 2005), potentially pointing to either greater exposures or higher deposition (excretion) rates, but dietary or other sources of Hg were not taken into account.

The CHARGE (CHildhood Autism Risks from Genetics and the Environment) Study is a large, comprehensive, epidemiologic investigation designed to identify factors associated with autism that may provide clues about etiology, comorbidity, or mechanisms of susceptibility. We measured and compared postdiagnosis blood Hg concentrations in preschool-age children with and without autism, adjusting for recent exposures through diet, personal care products, vaccines, and dental amalgams.

## Materials and Methods

### Design and data collection

The CHARGE Study is an ongoing population-based case–control investigation of three groups of children: with autism, with developmental delay (DD) but not autism, and from the general population. Autism and DD cases are recruited from the State of California Department of Developmental Services. Population-based controls are randomly selected from state birth files with frequency matching on age, sex, and broad geographic distribution of the autism cases. Eligibility criteria are age 24–60 months, living with at least one biological parent, born in California, and having a parent who speaks English or Spanish. All children are examined clinically, and diagnoses are confirmed following algorithms previously described (Hertz-Picciotto et al. 2006) based on administration of the Autism Diagnostic Observation Schedule (ADOS) (Le Couteur et al. 2003), Autism Diagnostic Inventory–Revised (ADI-R) (Lord et al. 1994), Mullen Scales of Early Learning (MSEL) (Mullen 1995), and Vineland Adaptive Behavior Scales (VABS) (Sparrow et al. 1984). After clinical evaluation, blood specimens are collected.

Trained bilingual/bicultural (Spanish-speaking) interviewers administer an extensive questionnaire by telephone to the parent. This instrument collects sociodemographic characteristics, occupational and residential histories, consumption of fish, personal care products, and medical and dental procedures and treatments. The respondent is mailed a calendar marked with key dates of her pregnancy to facilitate answering questions about timing of exposures.

Further study design and implementation details are available (Hertz-Picciotto et al. 2006). This study was approved by institutional review boards of the University of California–Davis School of Medicine and of the State of California and complied with all applicable U.S. requirements. All human participants gave written informed consent before data collection.

### Measurement of total Hg in blood

Blood samples were collected into EDTA and other tubes, transported to the laboratory and stored in −80°C freezers until subsampling of the 0.5 mL designated for metals analysis, which was then stored at −20°C. Analysis of total (inorganic + organic) Hg for eligible index children was carried out in batches of 30–40 between 2004 and 2006 using an Agilent 7500i (Agilent, Palo Alto, CA) inductively coupled plasma mass spectrometer in the Department of Civil and Environmental Engineering, University of California–Davis, with argon (Ar) plasma at 1,350 W. Samples were diluted 20-fold (200 μL + 3.8 mL) with water containing 1% nitric acid (trace metal grade; Fisher Scientific, Pittsburgh, PA), 0.05% Triton X-100 (Fisher), and 100 ppb terbium (Spex Certiprep, Methuen, NJ) as an internal standard. Uptake was 0.4 mL/min from a peristaltic pump with 1.2 L/min Ar carrier gas through a Babbington-style nebulizer into a Peltier-cooled double-pass spray-chamber at 2°C; 1 L/min auxiliary Ar and 12 L/min plasma gas Ar were added for a total of 14.2 L/min separated from nickel cones by a sampling depth of 8.5 mm. Oxides were tuned to < 0.4% cerium oxide/cerium (Ce) and double ions to 2% Ce^2+^/Ce^+^ to strengthen the signal for high ionization potential elements such as Hg. Sensitivity (counts per second/ppb) was tuned for high mass stability (relative SD < 3%) and sensitivity 20,000 for thallium at *m*/*z* = 205. Validation used the performance testing standards from New York State (Wadsworth Center, Albany, NY). Their targets are ± 20% for concentrations > 15 μg/L (ppb) ± 3 μg/L otherwise. Few of our samples exceeded 3 μg/L, which means they would meet criteria if all were reported as zero. Using 20 times the usual data acquisition for Hg, and 2.5 min of rinsing between samples, a detection limit of 0.02 μg/L was achieved.

### Exclusions

Among children enrolled from the fall of 2003 through 31 December 2006 in the CHARGE Study, blood Hg measurements were completed in 618. Of these, 344 met criteria on both ADOS and ADI-R for either autism or ASD (AU/ASD group), 68 were confirmed to have DD or atypical development without AU/ASD (met criteria for DD on at least one of the MSEL and VABS) and are designated DD, 166 recruited from the general population had confirmed typical development (TD), and 40 did not fall into any of these diagnostic classes or had incomplete assessment data and were excluded from further analyses. Further exclusion of 12 participants who had been chelated (mean blood Hg concentration = 0.13 μg/L) left 566. Of these, 31 were lacking complete interview information, 32 said they ate fish but answered no to each type of fish, 26 were interviewed > 90 days before the blood draw (behaviors might have changed), and 19 had missing values on one or more analysis variables; these 108 children and 6 siblings enrolled in the study were excluded. Analysis was conducted on the remaining 452 children: 249 AU/ASD, 143 TD, and 60 DD.

### Data analysis

Blood Hg concentrations were transformed using the natural logarithm because of the wide variation and skewed distribution. Nondetectable values were assigned the detection limit divided by 
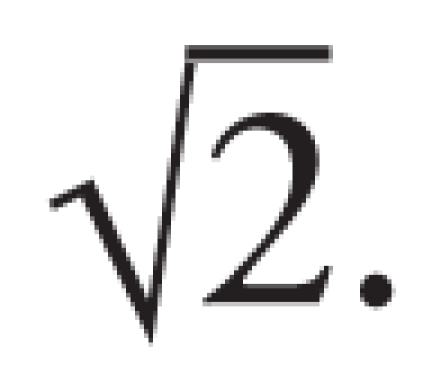
. Interview data were electronically captured using double entry with built-in consistency checks. The telephone interview asked about the mother’s and child’s fish consumption by type: tuna, other ocean fish, freshwater fish, and “fish that you caught or that someone else that you knew caught.” For these caught fish, we used the exact type of fish to categorize as tuna, freshwater, or ocean, but in some cases answers were inadequate for this determination. We therefore conducted sensitivity analyses by comparing results for four ways of handling caught fish of unknown type: *a*) assigning the ocean category, *b*) assigning the freshwater category, *c*) omitting consumption of the indeterminate caught fish, or *d*) excluding subjects with indeterminate type of fish. We also asked about frequency of consumption in a subset of parents and categorized total fish intake for 207 respondents by assigning for each type the number of fish meals per week [0 (none), 0.5 (< 1), 1.0 (1), and 2 (> 1)] and then summing across fish types, grouping the maximum category as two or more fish meals per week. Because the half-life of methylmercury in blood is measured in months, we focused on the child’s fish consumption during the current year of the child’s life, as reported in interviews conducted within 90 days of the blood draw.

Information on vaccines was obtained from the child’s vaccination card or pediatric medical record for 347 children, of whom 42 were immunized within 90 days of the blood draw. Hg doses were assigned based on dates of vaccine and types [for details, see Supplemental Material, Appendix I, available online (doi:10.1289/ehp.0900736.S1 via http://dx.doi.org)], and the contribution to blood Hg at the time of the blood draw was estimated assuming a 7-day half-life (Pichichero et al. 2002), although shorter half-lives have also been reported (Pichichero et al. 2008). Other possible sources of blood Hg in the child were obtained by interview with the mother, including dental amalgams and use of over-the-counter pharmaceutical products such as nasal sprays and earwax removal formulations. The interviewer asked: “Does [child’s name] have any dental amalgam (silver-colored) fillings?” and how many, as well as when they were placed. We used the number of amalgam fillings at the time of the interview for children who also either chewed gum or ground their teeth “a lot during the night or at other times” according to interview data. Nasal sprays and earwax removal products were combined into one variable, used or not used, during the year of the blood draw.

**Figure 1:**
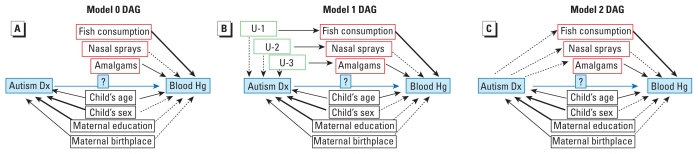
Directed acyclic graph (DAG) for models 0 (*A*), 1 (*B*), and 2 (*C*). Each arrow represents a cause-and-effect relationship: solid arrows, associations that are established, with heavier arrows indicating stronger associations; dotted arrows, speculative or weaker associations (e.g., without *a priori* evidence). Boxes labeled U-1, U-2, and U-3 in (*B*) represent hypothesized unmeasured confounders. The association we studied between autism diagnosis (Dx) and blood Hg concentration is represented with a question mark. Thimerosal-containing vaccines were omitted because of the small number exposed within the relevant time frame, but in each model in these DAGs this factor would have appeared identically as other Hg sources. For further explanation of these models, see “Materials and Methods.”

We conducted all analyses in SAS version 9.2 (SAS Institute Inc., Cary, NC). We compared children from the three confirmed diagnostic groups (AU/ASD, DD, and TD) with regard to demographic factors and sources of Hg. Each factor was evaluated as a determinant of blood Hg concentration in bivariate analysis. We then fitted several multiple linear regression models based on alternative sets of underlying causal relationships represented conceptually by directed acyclic graphs (Greenland et al. 1999) (Figure 1). To address confounding, we considered eight factors measured in this study potentially related to both the current blood Hg concentration and autism. Only six children were exposed to thimerosal-containing vaccines in the time period relevant for their respective blood Hg levels, rendering these analyses of low statistical power. For all models, except those with interaction terms, the assumption of standard multiple linear regression is that effects are homogeneous across strata of other variables.

In model 0 (Figure 1A), fish consumption, use of nasal sprays, and dental amalgams cause changes in child’s blood Hg level but have no association with autism diagnosis. Under this set of assumptions, analytic adjustment for these factors will not affect validity of the estimated association between autism diagnosis and blood Hg level.

In model 1 (Figure 1B), current fish consumption is influenced by an unmeasured earlier or preexisting causal factor associated with autism. For example, cultural, economic, or medical factors might be causally related to risk of autism and to current consumption of fish, dental amalgams, or use of nasal sprays. In this model, adjustment for current fish consumption, use of nasal sprays, and dental amalgams would be required to control confounding if their causal antecedents were not already controlled. Child’s sex and maternal education require control only if they contributed causally to a difference in blood Hg concentration or represented a surrogate that did.

In model 2 (Figure 1C), patterns of current fish consumption, use of nasal sprays, and presence of dental amalgams are assumed to be influenced by whether the child has autism. Children with autism reportedly have strong food preferences (Schreck et al. 2004) as well as sensory sensitivities (Rogers et al. 2003; Tomchek and Dunn 2007), supporting the arrows emanating from autism to these factors. If these factors are downstream of the autism phenotype, adjustment would be expected to bias estimates of the total effect of autism on blood Hg. In other words, if interest were in the total effect of an autism diagnosis, regardless of pathway or mechanism, the unadjusted analysis would be appropriate. Our interest, however, is in estimating the direct effect that is independent of fish consumption and other external sources of Hg and hence the analysis requires adjustment.

In a separate analysis, we applied methods to distinguish the impact of a causal factor through an intermediate versus a direct path (Petersen et al. 2006). This approach estimates the influence of diagnosis on blood Hg, independent of fish consumption; it is valid if all confounders of the associations between diagnosis and blood Hg and between fish consumption and blood Hg have been adjusted. The covariates we identified appear to satisfy this requirement.

Finally, building on model 1, we introduced interaction terms between diagnostic group and fish consumption, with the aim of determining whether the relationship between intake (exposure) and blood Hg was modified by diagnostic group. Conversely, we evaluated whether differences in blood Hg across diagnostic groups varied by fish consumption.

Mothers of participants in this study were more likely to be of higher socioeconomic status than those whom we attempted unsuccessfully to enroll (Table 1), especially among controls. We adjusted for differential participation with weights and a design effect, namely stratified sampling without replacement in SAS PROC SURVEYREG, in all regression analyses. We derived weights inversely proportional to the probability of participation by fitting a model for participation to the source population from which we recruited [see Supplemental Material, Appendix II (doi:10.1289/ehp.0900736.S1)].

## Results

Mothers of AU/ASD children closely resembled those of general population controls with regard to sociodemographics (Table 1). Mothers of DD children were less educated, younger, and less likely to have private health insurance. More DD children were females because we conducted no matching for this group.

**Figure 2:**
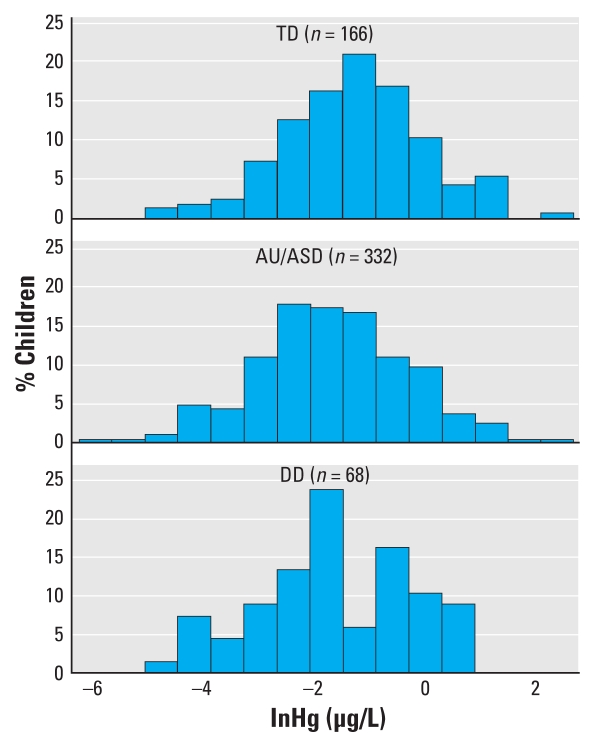
Distribution of the natural logarithm of total Hg concentrations in blood from children participating in the CHARGE study, 2003–2006 (*n* = 566), by diagnostic group: TD, AU/ASD, and DD.

In unadjusted analysis, TD children had higher means (Table 2) and overall distributions (Figure 2) of their blood Hg levels. The unadjusted geometric mean for TD children, 0.28 μg/L, was significantly higher than for AU/ASD (0.19 μg/L, *p* = 0.006) or DD (0.17 μg/L, *p* = 0.01) children. After adjustment for demographic factors and Hg sources and application of weights, the geometric means for AU/ASD, DD, and TD children were 0.26, 0.16, and 0.24 μg/L, respectively, with only the DD group significantly different from controls (*p* = 0.007). Overall, as well as within the AU/ASD and TD groups, those excluded (but not chelated) did not differ significantly in their blood Hg concentrations from those included in the analysis (geometric means for excluded vs. included, respectively, AU/ASD, 0.17 vs. 0.19 μg/L; TD, 0.27 vs. 0.28 μg/L). The excluded DD children (*n* = 8) had marginally higher blood Hg concentrations than those included (0.42 vs. 0.17 μg/L, *p* = 0.07).

We compared sources of Hg exposure across diagnostic groups (Table 2). Children with AU/ASD were less likely to consume tuna (*p* < 0.0001), other ocean fish (*p* < 0.0001), and freshwater fish (*p* < 0.0001). Compared with TD children, those with AU/ASD (*p* = 0.15) or DD (*p* = 0.13) were somewhat more likely to have used nasal sprays or earwax removal products. Thus, the assumptions of model 0—that fish and other sources of Hg do not differ between cases and controls—are violated, and model 0 was therefore rejected. The average thimerosal dose in the previous 90 days was not different for children with AU/ASD versus TD controls (*p* = 0.75).

Under model 1, which requires adjustment for fish consumption and other sources of Hg, children with AU/ASD had neither higher nor lower blood Hg concentrations than those from the TD group (β = 0.04, *p* = 0.75). DD children had a reduced blood Hg concentration relative to TD controls (β = −0.44, *p* = 0.03). The major predictors of blood Hg concentrations were consumption of tuna (β = 0.60, *p* < 0.0001), other ocean fish (β = 0.53, *p* < 0.0001), and freshwater fish (β = 0.70, *p* = 0.0006; Table 3). These results were robust to varying the assumptions about caught fish of indeterminate type (data not shown). A dose–response analysis (on those for which fish serving questions were asked) indicated a strong increase throughout the range from none to two or more fish meals per week (*p* < 0.0001), with coefficients of 0.67, 0.99, 1.40, and 1.83 in models of ln blood Hg (micrograms per liter) for 0.5, 1, 1.5, and ≥ 2 total fish meals per week, respectively. These translate into increases in blood Hg of 95%, 169%, 306%, and 523%, respectively. Additionally, higher blood Hg concentrations were found in children with dental amalgams who also grind their teeth or chew gum, compared with those who either have no amalgams or do not grind their teeth or chew gum (β = 0.17, *p* = 0.005). Use of nasal sprays or earwax removal products in the current year of the child’s life was not a significant predictor (β = 0.16, *p* = 0.33). Children whose mothers were born in neither the United States nor Mexico had higher blood Hg (β = 0.57, *p* = 0.0008). In an analysis limited to the 347 children for whom we obtained medical records on vaccines, those who received a thimerosal-containing vaccine within the previous 90 days did not have higher blood Hg levels (β = 0.01, *p* = 0.77).

Under model 2, which estimates the total effect of diagnostic group on blood Hg (including that mediated through behaviors that alter exposures to Hg), children with AU/ASD were found to have reduced blood Hg concentrations compared with TD controls (β = −0.34, *p* = 0.02), reflecting their lower fish consumption. Children with DD also had lower Hg levels, with the β-coefficient virtually unchanged from model 1 (β = −0.48, *p* = 0.03). Mother’s birthplace outside the United States and Mexico still predicted higher children’s Hg concentrations.

Because fish intake may be on the causal pathway, we applied specialized methods to calculate the direct effect of diagnosis on blood Hg (Petersen et al. 2006). Separate from any pathway mediated by variation in fish consumption, blood Hg was 12% lower in children with AU/ASD compared with TD controls.

Models with interaction terms demonstrated that the relationship of fish consumption (all types combined) to blood Hg did not differ by diagnostic group. When we stratified by fish consumption and compared AU/ASD children with TD controls, blood Hg levels differed neither among fish eaters (β = −0.002, *p* = 0.99) nor among non-fish eaters (β = −0.08, *p* = 0.73). Children with DD had marginally lower blood Hg than did TD controls among both those who ate fish (β = −0.38, *p* = 0.10) and those who did not (β = −0.67, *p* = 0.08). In contrast, chewing or grinding oral habits in the presence of amalgams resulted in increased blood Hg among TD controls (β = 1.17, *p* = 0.02) but not among the AU/ASD children (β = −0.13, *p* = 0.58).

## Discussion

The analyses presented here from CHARGE Study participants focus on blood Hg concentrations in children who have already been diagnosed with an ASD. They illuminate several facets of the blood Hg × AU/ASD relationships. First, fish consumption, a major contributor to human body burdens of methylmercury, was the primary predictor of total blood Hg in this population. Second, fish consumption was less prevalent in cases compared with controls. Consequently, when we ignored variation in fish intake (hence assuming it had no association with diagnosis), blood Hg in children with AU/ASD was significantly reduced compared with that of TD controls.

The third observation, however, is that after adjusting for dietary, medical, pharmaceutical, and dental sources of Hg, we found no difference in blood Hg comparing children with AU/ASD and TD controls, indicating that for a given exogenous exposure, cases did not differ from controls. Additionally, using specialized statistical methods to parse out the influence of behaviors that are affected by the autism diagnosis on blood Hg levels, we determined that, above and beyond the indirect impact on blood Hg mediated via fish intake, any direct effect of AU/ASD on blood Hg was small (~ 12% reduction). Finally, analyses restricted to non-fish-eating children demonstrated that cases had similar concentrations of blood Hg as did controls, after adjusting for non-fish Hg sources. Thus, three distinct analyses confirm that children with AU/ASD have blood Hg levels comparable with those of age-matched TD controls.

Notably, because half-lives of methylmercury in blood and whole-body inorganic Hg range from 60 to 90 days (Clarkson et al. 2003), these measurements cannot address whether Hg exposures in either the prenatal or early postnatal period play an etiologic role in autism. An alternate related hypothesis posits that children with autism have abnormal metabolism or inadequate excretion of Hg and that they sequester Hg in the brain, resulting in greater susceptibility to its developmental neurotoxicity. Because the present study used a single time measurement of Hg in blood, we could not directly evaluate excretion or determine tissue levels of Hg in the central nervous system. An autopsy study of general population adults showed strong correlations between methylmercury in blood and in the occipital cortex and between the number of dental amalgams and concentration of inorganic Hg in brain (Bjorkman et al. 2007). In monkeys, brain:blood ratios of total Hg were about 2.5 for those exposed to methylmercury (Burbacher et al. 2005). Because only 5% of body burdens of Hg are estimated to be in circulation (Burbacher et al. 2005; Stinson et al. 1989), reliable conclusions about distribution are not possible from one-time observational measurements in blood.

A further limitation was our lack of data on dietary factors other than fish, such as selenium or long-chain polyunsaturated fatty acids, which may mitigate effects of Hg (Beyrouty and Chan 2006; Ralston et al. 2008; Strain et al. 2008). Additionally, exposures to environmental Hg from ambient air pollution or other sources were not controlled in this analysis. Thus, our findings, that TD children with dental amalgams who chewed gum or ground their teeth had significantly higher blood Hg levels whereas AU/ASD children in that category did not, could be a result of unmeasured confounders that disproportionately affected AU/ASD children, or simply of the small number with this combined exposure (8 ASD, 4 DD, and 7 TD).

Strengths of this study include a relatively large sample size, recruitment of population-based cases and controls, and confirmation by trained clinicians of all diagnoses or lack thereof (in controls). The thorough collection of individual-level data with the assessment of multiple dietary, home, and medical sources of Hg is unique in the field of autism. Maternal reports of recent fish consumption were highly predictive of the child’s blood Hg level, suggesting strong validity of these questionnaire items. Further, we carried out a systematic approach to adjustment for selection bias, controlled confounding from numerous sociodemographic and other factors, and analyzed variability across developmental groups in blood Hg independently of fish intake. For these reasons, our results are likely to be both valid and generalizable.

In further support of generalizability, blood Hg values in these 2- to 5-year-old children with AU/ASD were similar to those reported for the U.S. NHANES nationally representative sample of 1- to 5-year-olds in 1999–2002 (Centers for Disease Control and Prevention 2004). In the NHANES sample, the geometric mean was 0.33 μg/L, the median 0.26 μg/L, and the interquartile range 0.10–0.61 μg/L (Centers for Disease Control and Prevention 2004); corresponding values in this study were 0.28, 0.23, and 0.10–0.57 μg/L (for this comparison, we used the NHANES detection limit of 0.14 to compute geometric means).

We presented several analyses because the underlying causal model is not certain. Current fish consumption and other Hg exposures might represent surrogates for earlier exposures or demographic factors that influence risk for AU/ASD or likelihood of diagnosis (Figure 1B), might be influenced by the child’s developmental status (Figure 1C), or both. Because our goal was to address whether children with a particular diagnosis exhibit differences in circulating Hg, adjustment for intake was essential (model 1, Table 3). This model confirmed no differences in blood Hg in AU/ASD versus TD controls.

That children with DD had lower concentrations of blood Hg, whether or not we adjusted for fish consumption or restricted analysis to non-fish eaters, may indicate either a chance finding in the smaller sample of DD children or selection bias beyond that associated with socioeconomic factors. Nevertheless, because metabolic disorders are known to accompany numerous developmental conditions, further research on Hg kinetics, including genetic and environmental influences, may be warranted.

Few studies on autism have considered dental amalgams, personal care products, or diet as sources of Hg. Two reports related autism risk to ambient Hg pollution as a potential etiologic factor (Palmer et al. 2006; Windham et al. 2006) but did not have individual-level dietary data. Similar to our finding, investigations of island populations (Grandjean et al. 1992) and the U.S. NHANES data (Mahaffey et al. 2004) have reported fish and seafood consumption to be a major contributor to Hg measured in biospecimens. In a longitudinal study of 67 persons with initially elevated blood or hair Hg, reduction in fish consumption was followed by a steep decline in blood Hg level (Hightower and Moore 2003). In nursing mothers from Germany, breast milk concentrations of Hg were strongly influenced by fish consumption, and maternal amalgams were a major source of infant Hg shortly after birth (Drexler and Schaller 1998). A study in eastern Slovakia, where fish consumption is very low, demonstrated higher cord blood Hg concentrations with a greater number of maternal Hg amalgams, or a shorter time since the most recent filling; by 6 months of age, maternal amalgams were no longer determinants of the infant’s total blood Hg (Palkovicova et al. 2008). Exposure from Hg amalgams occurs through inhalation of vapor released into the oral cavity, and both organic and inorganic Hg concentrations in saliva (Leistevuo et al. 2001) and urinary Hg (Hansen et al. 2004) are elevated in direct relation to the number of amalgam tooth surfaces.

The strong associations of maternally reported fish consumption and dental amalgams with blood Hg suggest that these questionnaire items provided valid information on recent exposures. Three types of fish each contributed independently to blood Hg. Interestingly, although few children had Hg amalgams, those who did and who also either chewed gum or had bruxism appeared to have experienced sufficient release of inorganic Hg to be measurable in blood. This finding is consistent with a report on adult chewers of nicotine gum (Sallsten et al. 1996) and an investigation of amalgam Hg releases into chewing gum (Hansen et al. 2004). Use of two over-the-counter thimerosal-containing products—nasal sprays and earwax removal formulations—was initially suggestively associated with elevated blood Hg but not in fully adjusted models. Misclassification of this exposure (relative to the timing of the blood draw) was possible because these products tend to be used intermittently and because some may not contain thimerosal.

The higher blood Hg in children whose mothers were born in neither the United States nor Mexico might have arisen from greater seafood consumption. These mothers came from all over the world. In the early phase of the study, we did not obtain the amount and timing of consumption of seafood, which contains per weight Hg levels about one-fourth those in fish (Mahaffey et al. 2004).

## Conclusion

Children 2–5 years of age with autism or other ASD had blood Hg concentrations similar to those of TD controls after adjustment for a variety of home and medical Hg sources. This finding was maintained when analysis was restricted to non-fish eaters. Blood Hg levels in both controls and cases were very close to those of a nationally representative sample of 1- to 5-year-olds in the United States.

The primary limitation in this study is the lack of longitudinal measurements that could address toxicokinetics. Even with a one-time measurement, however, this case–control study still represents the most rigorous examination to date of differences in circulating blood Hg associated with autism. Numerous strengths distinguish this from previous reports, such as confirmation of diagnoses; collection of detailed data on multiple dietary, home, and medical sources of Hg; control of numerous confounders; and statistical adjustment for self-selection toward more highly educated parents, especially in population-based controls. These aspects of the design, data collection, and analysis support validity and generalizability of the results.

This report did not address the role of prenatal or early-life Hg exposures in etiology of autism. Analysis of specimens that predate the autism diagnosis would be required to answer that question.

## Figures and Tables

**Table 1:** Demographic characteristics of CHARGE Study children with Hg analysis^a^ and of the CDDS target population, by case group, California, 2003–2006 (%).

	GP controls		AU and ASD cases		DD cases	
Characteristic	Participants with TD (*n* = 166)	GP target population (*n* = 2,557)	Participants with confirmed AU or ASD (*n* = 332)	CDDS target population of AU (*n* = 2,101)	Participants with confirmed DD (*n* = 68)	CDDS target population of DD (*n* = 804)
Mothers ≥ 35 years of age at delivery	25	16	24	23	19	22
Mother’s education at delivery (years)						
< 12	7	27	5	14	19	27
≥16	52	26	45	32	22	18
Mother’s birthplace						
USA	79	57	76	61	68	63
Mexico	7	21	9	15	22	21
Neither USA nor Mexico	14	21	15	24	10	16
Payment method for delivery						
Public	13	38	16	27	35	47
Private	87	62	84	73	65	53
Sex: male child	81	81^b^	89	82	71	65
Age (years)						
2	39	—c	21	—c	25	—c
3	36		38		42	
4	25		41		33	

Abbreviations: AU, autism; CDDS, California Department of Developmental Services; GP, general population.

a Excluding chelated subjects.

b Sex distribution in the GP pool is skewed because of frequency matching.

c The age distribution of the target population was changing throughout the study, as children aged.

**Table 2:** Blood Hg concentration and potential sources of Hg by diagnostic group (unweighted), CHARGE Study children 2–5 years of age, California, 2003–2006.

Characteristic	TD (*n* = 143)	AU/ASD (*n* = 249)	DD (*n* = 60)
Mean ± SD			
Hg	0.60 ± 1.03	0.49 ± 1.08	0.39 ± 0.51
Ln(Hg)	−1.28 ± 1.26	−1.67 ± 1.37	−1.77 ± 1.40
Hg [geometric mean (geometric SD)]^a^	0.28 (3.53)	0.19 (3.94)	0.17 (4.05)
Estimated thimerosal dose^b^ from vaccine^c^	0.06 ± 0.51	0.04 ± 0.54	0.01 ± 0.03
Percent			
Dental amalgams and chew or grind teeth	5	3	8
Thimerosal-containing vaccine (previous 90 days)^c^	1.9	1.5	2.4
Ate any fish	76	43	68
Ate tuna	44	18	43
Ate ocean fish	58	36	48
Ate freshwater fish	20	6	15
Used nasal spray or earwax removal product	13	19	23

Values are mean ± SD, geometric mean (SD), or percent.

a After adjustment for demographic factors and Hg sources and application of weights, the geometric means for TD, AU/ASD, and DD children were 0.24, 0.26, and 0.16 μg/L, respectively.

b Assumes 7-day half-life in blood; calculation based on vaccines in previous 90 days.

c Because of missing information on vaccines, total *n* = 347 (106 TD, 199 AU/ASD, and 42 DD).

**Table 3:** Multiple linear regression models predicting ln blood Hg level in CHARGE Study children 2–5 years of age to California to 2003–2006.

	Model 1			Model 0 or model 2		
Characteristic	β-Coefficient (SE)	*p*-Value	Predicted percent change in blood Hg (95% CI)	β-Coefficient (SE)	*p*-Value	Predicted percent change in blood Hg (95% CI)
Developmental diagnosis						
TD (reference)	—			—		
AU or ASD	0.04 (0.14)	0.75	4 (−21 to 37)	−0.34 (0.14)	0.02	−29 (−46 to −6)
DD or atypical	−0.44 (0.20)	0.03	−36 (−56 to −5)	−0.48 (0.22)	0.03	−38 (−60 to −5)
Mother’s education (years)						
< 12 (reference)	—			—		
12–15	−0.21 (0.21)	0.31	−19 (−46 to 22)	−0.23 (0.25)	0.35	−21 (−51 to 30)
≥16	−0.09 (0.22)	0.68	−9 (−41 to 41)	−0.19 (0.25)	0.46	−17 (−49 to 35)
Mother’s birthplace						
USA (reference)	—			—		
Mexico	0.09 (0.21)	0.67	9 (−28 to 65)	−0.06 (0.26)	0.82	−6 (−43 to 57)
Neither USA nor Mexico	0.57 (0.17)	0.0008	77 (27 to 147)	0.77 (0.17)	< 0.0001	116 (55 to 201)
Child’s age (years)	−0.03 (0.08)	0.66	−3 (−17 to 14)	0.01 (0.08)	0.92	1 (−14 to 18)
Child’s sex (female vs. male)	0.19 (0.16)	0.22	21 (−12 to 65)	0.29 (0.16)	0.07	34 (−2 to 83)
Ate tuna (yes vs. no)	0.60 (0.14)	< 0.0001	82 (38 to 140)			
Ate ocean fish (yes vs. no)	0.53 (0.13)	< 0.0001	70 (32 to 119)			
Ate freshwater fish (yes vs. no)	0.70 (0.20)	0.0006	101 (36 to 198)			
Hg amalgams in gum chewers or teeth grinders (per amalgam)	0.17 (0.06)	0.0052	19 (5 to 33)			
Nasal spray or earwax removal use (yes vs. no)	0.16 (0.17)	0.33	17 (−16 to 64)			

AU, autism.
